# Evaluating the Knowledge and Practices of General Practitioners in Cardiovascular Risk Assessment at Three Referral Hospitals in Bujumbura, Burundi

**DOI:** 10.24248/eahrj.v9i1.833

**Published:** 2025-09-30

**Authors:** Zacharie Ndizeye, Ghislain Mutwenzi, Désiré Habonimana, Jean Claude Nkurunziza, Sandra Nkurunziza, Elysée Baransaka

**Affiliations:** a Department of Community Medicine, Faculty of Medicine, University of Burundi, Bujumbura, Burundi; b Centre Hospitalo-Universitaire de Kamenge (CHUK), Bujumbura, Burundi; c Department of Internal Medicine, Faculty of Medicine, University of Burundi, Bujumbura, Burundi

## Abstract

**Background:**

Cardiovascular diseases (CVD) encompass a range of non-communicable conditions that share a common pathophysiological process related to atherosclerosis. Currently, CVD are the leading cause of death worldwide, with an estimated 17.9 million deaths attributed to these conditions in 2019. In Africa, the burden of CVD is steadily increasing, leading to a rise in years lived with disability. In Burundi, however, cardiovascular diseases are poorly documented, and there is a lack of data on the extent to which general practitioners (GPs) assess cardiovascular risk among their patients. This study aimed to evaluate the knowledge and practices of GPs regarding global cardiovascular risk assessment, providing evidence to inform policymakers and training institutions on potential strategies for improving cardiovascular care.

**Methods:**

A descriptive, cross-sectional study was conducted in three national hospitals in Burundi: Centre Hospitalo-Universitaire de Bujumbura (CHUK), Hôpital Prince Régent Charles (HPRC), and Hôpital Militaire de Kamenge (HMK), all located in Bujumbura capital city. Study participants were general practitioners working in the outpatient departments of these hospitals. Data were collected using a validated questionnaire, and descriptive statistics were generated to analyse the findings. The level of knowledge regarding cardiovascular disease was assessed based on scoring systems derived from the questionnaire. The study received ethical approval from the Institutional Review Board of the Faculty of Medicine, University of Burundi.

**Results:**

The participation rate was 86.8%, with 66 participants out of 76 completing the study. Among respondents, 68% had less than five years of professional experience. Only 40.9% demonstrated adequate knowledge of cardiovascular disease, and a mere 18.18% possessed sufficient knowledge of therapeutic objectives and treatment strategies. In practice, 77.3% of participants reported routinely assessing cardiovascular risk; however, 42% of those in need of lipid-lowering therapy did not prescribe such medications, highlighting gaps between clinical practice and guideline adherence

**Conclusions:**

Identified gaps in the knowledge, attitudes, and practices of general practitioners concerning cardiovascular disease prevention highlight the need for decision-makers, training institutions, and universities to prioritise the development of ongoing in-service training programmes to enhance their capacity in this area.

## BACKGROUND

Cardiovascular diseases (CVD) comprise a group of diverse non-communicable conditions affecting the heart and blood vessels, primarily linked to atherosclerosis, the accumulation of fatty deposits within the arteries.^1^ These pathologies are a major contributor to global morbidity and mortality, posing a significant public health challenge. Currently, CVD is the leading cause of death worldwide; in 2019, an estimated 17.9 million deaths were attributed to CVD, accounting for approximately 32% of all global fatalities.^2^

In Africa, the burden of cardiovascular disease (CVD) is steadily rising, leading to an increase in years lived with disability.^3^ Globally, over three-quarters of CVD-related deaths occur in low- and middle-income countries that often lack effective, integrated primary healthcare programmes to identify and manage at-risk populations.^2,3^ The incidence of CVD is expected to continue increasing, driven not only by the growing prevalence of obesity, diabetes, and metabolic syndrome but also by overall increases in life expectancy, including in developing countries. This demographic shift reflects an ageing population, which further amplifies the healthcare challenge associated with CVD.^4^

In Burundi, CVD are poorly documented, with estimated mortality rates around 10%.^5^ A 2012 study examining consultation reasons at the “Maison Médicale de Bujumbura” cardiology clinic found that arterial hypertension, cardiomyopathy, and valvulopathy were the most common diagnoses, accounting for 61.4%, 12.8%, and 10.2% of cases, respectively.^6^ Additionally, the 2020 Burundi statistical yearbook indicates that hypertension ranks 19^th^ among causes of hospital mortality.^7^ A retrospective study conducted in 2016 at the University Teaching Hospital (Centre Hospitalo-Universitaire de Kamenge, CHUK) revealed that patients with CVD comprised approximately 7% of all inpatients, underscoring the significance of CVD as a public health concern in Burundi.^8^ Effective prevention of CVD involves addressing and reducing its various risk factors, which should be implemented both at the individual and population levels.^9^ Prevention efforts require a comprehensive understanding of cardiovascular risk factors, as well as adherence to hygiene and dietary recommendations, particularly among healthcare providers responsible for maintaining and restoring health. Several tools, such as the Framingham score, Risk Factor Summation, NHANES score, SCORE, and MESA score; are widely used in routine clinical practice to assess cardiovascular risk, guide management, and improve patient outcomes. Ideally, all general practitioners should utilize these tools to identify and manage cardiovascular risk factors. However, there is currently no data available on the extent to which general practitioners (GPs) in Burundi evaluate cardiovascular risk among their patients. This study aimed to assess the knowledge and practices of general practitioners regarding comprehensive cardiovascular risk assessment. The findings will provide valuable insights for policymakers and medical training institutions, guiding targeted interventions and training programmes to enhance cardiovascular disease prevention in Burundi.

## METHODS

### Study Design, Location and Time

The study employed a descriptive cross-sectional design conducted across three national hospitals in Burundi: Centre Hospitalo-Universitaire de Bujumbura (CHUK), Hôpital Prince Régent Charles (HPRC), and Hôpital Militaire de Kamenge (HMK), all located in Bujumbura city. Burundi's health system is organized as a pyramidal structure comprising four levels: the central, intermediate, peripheral, and community levels, which are interconnected through hierarchical relationships.^10^ Within this framework, the national hospitals occupy the apex of the pyramid and provide specialized services. There are five national hospitals in Burundi, all situated in Bujumbura, but the selection of these three hospitals was based on their high patient volume and the frequency of recorded cardiovascular pathologies, as well as the availability of logistical resources at the time of the study. Data collection was carried out over a period of four months, from June 27 to October 30, 2020.

### Study Population and Inclusion Criteria

All general practitioners working in the outpatient departments of the three selected hospitals were eligible for the study. However, only those who consented to participate were included in the study.

### Data Collection Instruments and Procedures

The questionnaire was developed by the research team and validated by two cardiologists and four epidemiologists. It was pretested with five general practitioners at Prince Louis Rwagasore Hospital (not part of the study). Results from the pretest were used to rephrase and clarify some ambiguous questions. After compiling a complete list of GPs in the three selected hospitals, each participant received an anonymous, self-administered questionnaire, accompanied by an explanation of the study's objectives. Participants could complete the questionnaire immediately in the presence of the surveyor or schedule an appointment for collection after filling it out. The questionnaire included items on sociodemographic characteristics, health status, habits and lifestyle, recent biological monitoring, knowledge of cardiovascular risk factors, protective factors, diseases, risk assessment methods, treatment and prevention strategies, anthropometric measurements, and GPs’ practices related to cardiovascular risk prevention.

### Data Processing and Analysis

Data were entered into Microsoft Excel 2010 and imported into the IBM software, Statistical Package for the Social Sciences (SPSS), version 20 (IBM Corp, Armonk, NY, USA) for analysis. The analysis began with descriptive statistics of the population based on various characteristics, expressed as percentages. To evaluate knowledge levels, four scores were created from selected items: (a) knowledge of cardiovascular diseases (9 questions), (b) knowledge of risk and protective factors (18 questions), (c) understanding of individuals needing cardiovascular risk assessment (4 questions), and (d) knowledge of treatment objectives and prevention strategies (11 questions). Each correct answer scored one point; incorrect answers scored zero. Scores were calculated as the percentage of correct answers, with a threshold of 70% or higher indicating sufficient knowledge.

### Administrative and Ethical Considerations

Before the survey, approval was obtained from the Institutional Review Board of the Faculty of Medicine, University of Burundi (Reference number: FM/CE/02/09/2020), and permissions were secured from each hospital's administration. All participants provided voluntary oral consent, and personal data were kept confidential, used anonymously, and were accessible only to the researchers.

## RESULTS

### Socio-Demographic Characteristics of Participants

During the study, 76 general practitioners were assigned to the outpatient departments of three referral hospitals, out of whom 66 participated, resulting in an 86.8% participation rate. The mean age was 33.4 years (SD: ±3.07), with the 25–35 years age group being the most common. Males accounted for 56.06%. Participants had an average of 3.6 years (SD: ±2.22) of professional experience, with 68% having less than five years. Most had a normal Body Mass Index (BMI), did not consume alcohol or smoke, and had no major CVD history (Table 1).

**TABLE 1:** Socio-Demographic Characteristics of Participants (N=66)

Variables	n (%)
Age in years	
<25	1 (1.5)
25–35	51 (77.3)
36–45	14 (21.2)
Average age (SD)	33.4 (±3.1)
Gender	
Male	37 (56.1)
Female	29 (43.9)
Years of practice	
< 5 years	45 (68.2)
6–10 years	20 (30.3)
> 10 years	1 (1.5)
Average (SD)	3.63 (±2.2.)
BMI	
Between 18–24.9 (normal)	45 (68.2)
Between 25–29.9 (overweight)	19 (28.8)
Over 30 (obesity)	2 (3.0)

Abbreviation: SD, standard deviation; BMI, body mass index.

### Participants’ Knowledge and Practices

Only 40.9% of participants demonstrated sufficient general knowledge of CVD (score ≥70%), with 26% being aware of its global mortality ranking, and 54% and 65% recognizing heart defects and deep vein thrombosis respectively as CVD (Table 2).

**TABLE 2:** General Knowledge of Cardiovascular Diseases (N=66)

General knowledge	Correct Answer n (%)	Sufficient Level of Knowledge (Score ≥70%)
**Place of CVD mortality in the world**	**17 (25.8)**	**27 (40.9%)**
CVD		
Aortic Aneurysm	40 (60.6)	
Arteriopathies of the lower limbs	57 (86.4)	
Stroke	65 (98.5)	
Rheumatic heart disease	41 (62.1)	
Pulmonary embolism	41 (62.1)	
Coronary heart disease	65 (98.5)	
Cardiac malformations	36 (54.5)	
Deep vein thrombosis	43 (65.2)	

Abbreviation key: CVD: Cardiovascular Diseases.

Knowledge of CVD risk and protective factors was excellent (100% of GPs scored above 70%) (Table 3), but only 18.2% had sufficient knowledge of therapeutic objectives and treatments.

**TABLE 3:** Knowledge of Cardiovascular Diseases Risk and Protective Factors (N=66)

Associated Factors	Correct Answer n (%)	Sufficient Level of Knowledge ≥70%
**Risk factors for cardiovascular diseases**		66 (100)
Advanced age	66 (100)	
Diet high in saturated fats	66 (100)	
Family history of early-cardiovascular disease	66 (100)	
Diabetes	66 (100)	
Dyslipidemias	66 (100)	
Arterial hypertension	66 (100)	
Menopause	52 (78.8)	
Obesity/Overweight	66 (100)	
Sedentary lifestyle	66 (100)	
Gender	39 (59.1)	
Stress	66 (100)	
Metabolic syndrome	63 (95.4)	
Tobacco	66 (100)	
**Protective factors**		
Regular physical activity	65 (98.5)	
Healthy diet rich in dietary fiber & antioxidants (vegetables, fruits)	66 (100.0)	
Moderate alcohol consumption	39 (59.1)	
Low-dose Aspirin for cardiovascular risk factors)	64 (96.9	
High levels of HDL cholesterol	28 (42.4)	

Abbreviation: HDL: High density lipid

Most (87.8%) understood the profile of individuals needing cardiovascular risk assessment (Table 4), though familiarity with assessment methods was limited, only the summation of risk factors was well known (most familiar), while other tools like Framingham, SCORE, NHANES, and MONICA scores were poorly recognized (24.4%, 10.6%, 12.1%, and 4.5%, respectively) (Table 5).

**TABLE 4:** Knowledge of Cardiovascular Risk Prevention and patient profiles for Assessment, Objectives and Treatment Options (N=66)

Therapeutic Goals	Correct Answer n (%)	Overall Sufficient Level of Knowledge (≥70%)
**Blood pressure**		12 (18.2%)
Individuals without risk factor (BP<140/90 mmHg)	37 (56.1)	
Individuals with risk factor (BP<130/80 mmHg)	44 (66.7)	
**Total cholesterol**		
Individuals with no risk factor (<5 mmol/l or <1.9 g/l	16 (24.2)	
**Blood pressure**		12 (18.2%)
Individuals without risk factor (BP<140/90 mmHg)	37 (56.1)	
Individuals with risk factor (BP<130/80 mmHg)	44 (66.7)	
**Total cholesterol**		
Individuals with no risk factor (<5 mmol/l or <1.9 g/l	16 (24.2)	
Individuals with risk factor (<4.5 mmol/l or <1.75 g/l)	15 (22.7)	
**LDL-cholesterol**		
Individuals with no risk factor (<3 mmol/l or <1.15 g/l	9 (13.6)	
Individuals with risk factor (<2.5 mmol/l or <1.0 g/l)	17 (25.7)	
**Blood glucose**		
Individuals with no risk factor	46 (69.7)	
Individuals with risk factor	36 (54.5)	
Recommended BMI (<25 kg/m^2^)	61 (92.4)	
Physical activity (≥ 30 minutes/day)	64 (96.9)	
Therapeutic means	49 (74.2)	
**Profile of people for whom CVR assessment is recommended**		58 (87.8)
Anyone who requests it	32 (48.5)	
Anyone with one or more risk factor	66 (100)	
Anyone with a family history of cardiovascular disease or risk factor	61 (92.4)	
Anyone with symptoms suggestive of cardiovascular disease	58 (87.9)	

Abbreviations: BP: Blood pressure; LDL: Low-density lipoprotein; CVR: Cardio-vascular risks; BMI: Body mass index

**TABLE 5:** General Practitioners’ Practices (N=66)

Practices	n (%)
Practice of the sport activity	
Moderate (walking, cycling)	9 (13.6)
Intense (jogging, basketball, soccer, swimming)	34 (51.5)
No sports activities	23 (34.8)
Blood pressure control regularly	
Yes	28 (42.4)
No	38 (57.6)
Frequency of BP measurement	
At least once a week	3 (4.5)
At least once a month	14 (21.2)
At least once a quarter	9 (13.6)
At least once a semester	1 (1.5)
More than six months	1 (1.5)
No regular measurements	38 (57.6)
Regular biological check-up	
Yes	20 (30.3)
No	46 (69.7)
Frequency of regular biological check-ups	
At least once a quarter	5 (7.6)
At least once a semester	2 (3.0)
At least once a year	13 (19.7)
Irregular or more than one year	46 (69.7)
Biological check-up in the last six months	
Total cholesterol	14 (21.2)
Fasting blood glucose	34 (51.5)
HDL-Cholesterol	11 (16.7)
LDL-Cholesterol	11 (16.7)
Triglycerides	11 (16.7)
Information for at-risk patients	
Always	25 (37.9)
Often	39 (59.1)
Rarely	2 (3.0)
Never	0 (0)
Cardiovascular risk assessment	
Always	12 (18.2)
Often	39 (59.09)
Rarely	10 (15.1)
Never	5 (7.6)
Methods used to evaluate the CVR	
Summation of risk factors	60 (90.9)
Framingham score	1 (1.5)
SCORE score	0 (0)
NHANES score	0 (0)
MONICA score	0 (0)
No	5 (7.6)
Prescription of lipid-lowering drugs as a preventive measure	
Always	10 (15.1)
Often	28 (42.4)
Rarely	25 (37.9)
Never	3 (4.5)
Prescription of antiplatelet agents as a preventive measure	
Yes	61 (92.4)
No	2 (3.0)
Don't know	3 (4.5)
Proposal of lifestyle and dietary measures to patients	
Always	33 (50.0)
Often	29 (43.9)
Rarely	4 (6.1)
Never	0 (0.0)

Abbreviations: HDL: High-density lipoprotein; LDL: Low-density lipoprotein; CVR: Cardio-vascular risks; CVD: Cardio-vascular disease.

Practically, 35% did not engage in sports, 58% rarely checked their blood pressure, and 70% seldom monitored their biological tests (Table 5). While 77.3% reported regularly assessing clients’ cardiovascular risk, 42% rarely or never prescribed lipid-lowering drugs to those in need, and 7.6% were unaware of the importance of antiplatelet therapy. Only half consistently advised clients on dietary measures.

## DISCUSSION

Our participants exhibited a high response rate, surpassing similar studies,^11,12^ and this is the first known assessment in Burundi of GPs’ knowledge and practices regarding global CVR evaluation. The study revealed a low level of knowledge among GPs about CVD and their mortality impact, consistent with findings from other countries.^4,13,14^ While all participants demonstrated adequate understanding of CVD risk and protective factors, fewer than 20% had sufficient knowledge of therapeutic goals and treatment options. This gap is concerning, given GPs’ frontline role in CVD management in Burundi, where cardiologist shortages are prevalent. The insufficient knowledge likely stems from gaps in basic medical training and limited continuing education, highlighting the urgent need for targeted awareness and training to improve patient management.

In addition, practice in CVR assessment was inadequate: only 38% of GPs regularly inform patients about their cardiovascular risk, and just 18% always perform risk assessments. Effective doctor-patient communication is essential for better management, engagement, and adherence, yet poor CVR assessment practices undermine early detection of high-risk individual.^13,15^ Similar deficiencies have been documented elsewhere,^4,16^ with mixed findings on the influence of practitioner seniority.^4,17^ Furthermore, routine CVR assessment remains suboptimal, with approximately 90% of participants providing hygiene and dietary advice, half consistently and 43.94% often, but less than half prescribing lipid-lowering medications for high-risk or existing CVD patients, and 4.55% never do it. This indicates a gap in preventive management, as evidence supports the use of statins in high-risk individuals to reduce CVD incidence.^18-20^

### Study Limitations

The questionnaires were administered to participants, with some being collected later. In addition, participants were allowed to consult any relevant documentation while answering, which may have led to an overestimation of their knowledge of certain items. Furthermore, since participants were not selected randomly, the extent to which these findings can be generalised to a broader population is limited.

## CONCLUSION

Significant gaps in the knowledge and practices of Burundian general practitioners regarding CVD prevention highlight the urgent need for ongoing medical education and curriculum reform to enhance their skills in cardiovascular disease management.
